# Meningioma Surgery in Patients ≥70 Years of Age: Clinical Outcome and Validation of the SKALE Score

**DOI:** 10.3390/jcm10091820

**Published:** 2021-04-22

**Authors:** Daniel Monden, Florian J. Raimann, Vanessa Neef, Daniel Dubinski, Florian Gessler, Fee Keil, Marie-Thérèse Forster, Michael W. Ronellenfitsch, Patrick N. Harter, Thomas M. Freiman, Elke Hattingen, Volker Seifert, Christian Senft, Peter Baumgarten

**Affiliations:** 1 Department of Neurosurgery, University Hospital Frankfurt, Goethe University, 60528 Frankfurt, Germany; Daniel.Monden@med.uni-jena.de (D.M.); daniel.dubinski@med.uni-rostock.de (D.D.); florian.gessler@med.uni-rostock.de (F.G.); marie-therese.forster@med.uni-frankfurt.de (M.-T.F.); Thomas.Freiman@med.uni-rostock.de (T.M.F.); v.seifert@em.uni-frankfurt.de (V.S.); christian.senft@med.uni-jena.de (C.S.); 2 Department of Anaesthesiology, Intensive Care Medicine and Pain Therapy, University Hospital Frankfurt, Goethe University, 60528 Frankfurt, Germany; Florian.Raimann@kgu.de (F.J.R.); Vanessa.Neef@kgu.de (V.N.); 3 Institute of Neuroradiology, University Hospital Frankfurt, Goethe University, 60528 Frankfurt, Germany; Fee.Keil@kgu.de (F.K.); elke.hattingen@kgu.de (E.H.); 4 Department of Neuro-Oncology, University Hospital Frankfurt, Goethe University, 60528 Frankfurt, Germany; Michael.Ronellenfitsch@kgu.de; 5 Neurological Institute (Edinger-Institute), University Hospital Frankfurt, Goethe University, 60528 Frankfurt, Germany; patrick.harter@kgu.de; 6 German Cancer Consortium (DKTK) partner site, 60590 Frankfurt am Main, Germany; 7 German Cancer research Center (DKFZ), 69120 Heidelberg, Germany; 8 Frankfurt Cancer Institute (FCI), University Hospital Frankfurt, Goethe University, 60528 Frankfurt, Germany

**Keywords:** meningioma, elderly, KPS, SKALE score

## Abstract

Along with increasing average life expectancy, the number of elderly meningioma patients has grown proportionally. Our aim was to evaluate whether these specific patients benefit from surgery and to investigate a previously published score for decision-making in meningioma patients (SKALE). Of 421 patients who underwent primary intracranial meningioma resection between 2009 and 2015, 71 patients were ≥70 years of age. We compared clinical data including World Health Organization (WHO) grade, MIB-1 proliferation index, Karnofsky Performance Status Scale (KPS), progression free survival (PFS) and mortality rate between elderly and all other meningioma patients. Preoperative SKALE scores (Sex, KPS, ASA score, location and edema) were determined for elderly patients. SKALE ≥8 was set for dichotomization to determine any association with outcome parameters. In 71 elderly patients (male/female 37/34) all data were available. Postoperative KPS was significantly lower in elderly patients (*p* < 0.0001). Pulmonary complications including pneumonia (10% vs. 3.2%; *p* = 0.0202) and pulmonary embolism (12.7% vs. 6%; *p* = 0.0209) occurred more frequently in our elderly cohort. Analyses of the Kaplan Meier curves revealed differences in three-month (5.6% vs. 0.3%; *p* = 0.0033), six-month (7% vs. 0.3%; *p* = 0.0006) and one-year mortality (8.5% vs. 0.3%; *p* < 0.0001) for elderly patients. Statistical analysis showed significant survival benefit in terms of one-year mortality for elderly patients with SKALE scores ≥8 (5.1 vs. 25%; *p* = 0.0479). According to our data, elderly meningioma patients face higher postoperative morbidity and mortality than younger patients. However, resection is reasonable for selected patients, particularly when reaching a SKALE score ≥ 8.

## 1. Introduction

Meningiomas are among the most common intracranial tumors, accounting for about one third of all CNS-tumors [1]. Multiple epidemiological studies have shown an increasing incidence of meningioma, especially in the elderly population [2,3,4]. In addition, large-scaled autopsy studies pointed to a peak incidence of meningioma occurrence in the seventh to eighth decade of life and a rate of incidental meningioma of 2.3% [5,6]. With regard to histopathological grading, Park et al. reported an increased rate of WHO grade II and III meningiomas in patients >60 years of age [7].

Despite increased incidence rates and a higher proportion of aggressive tumors, there is no valid therapy recommendation for older patients. Currently, the majority of older meningioma patients are treated surgically, although promising alternatives such as conservative management or stereotactic radiotherapy are warranted at least in selected cases [8,9]. Several studies have already addressed the question of whether elderly patients benefit from surgical intervention with partially conflicting results [10,11,12]. The age-cutoff to define “elderly” patients was mostly set at 60–65 years of age, which might be too young considering an increasing life expectancy [11,12,13,14,15].

Although different scoring systems to identify patients who are at risk or might benefit from surgery have been proposed, none of them is widely used in clinical practice so far [16,17,18,19,20]. In 2013, Konglund et al. compared several prognostic systems for patients between 80 and 90 years of age and concluded that SKALE score might provide the most accurate prediction for the postoperative outcome [21]. This score was introduced by Sacko et al. in 2007 and awards a score between 0 and 16 based on the parameters sex, Karnofsky performance status scale (KPS), American society of anesthesiology score (ASA), location of tumor and peritumoral edema, with the recommendation for surgery on patients with a value ≥8 [16].

The aim of our monocentric study was to report on the outcome of elderly patients, whom we defined as patients ≥70 years of age and to compare main outcome parameters in this subgroup to our younger cohort. Furthermore, we aimed to evaluate the SKALE score as a predictive marker for outcome in the elderly population.

## 2. Materials and Methods

### 2.1. Patients

A total of 421 patients who underwent meningioma resection at our institution between 2009 and 2015 were retrospectively evaluated. Inclusion criteria was first surgery of an intracranial meningioma, while spinal manifestations or recurrences of a previously surgically treated intracranial meningioma were excluded from our cohort. 

### 2.2. Evaluation of Clinical Data

Patient treatment and survival data were collected in an institutional tumor registry. If not specifically noted, additional clinical data were retrospectively extracted from the electronic patient records. These included physician’s letters, histological reports, surgery reports and anesthesiologic protocols. If not entered in our tumor registry, survival data were obtained from public registration offices following individual inquiries. The ASA score was documented by the Department of Anesthesiology during the preoperative examinations [22]. The results of the primary MRI images were retrospectively evaluated by an experienced neuroradiologist.

Primary assessment of the KPS was performed by a neurosurgical resident and a responsible senior physician at first presentation in our outpatient clinic [23]. Postoperative KPS was collected at discharge by resident, senior physician and nursing staff, while last contact KPS was determined at last presentation in our outpatient clinic.

Measurement of tumor volume was performed using the SmartBrush tool of the Brainlab Elements software (Brainlab AG, Munich, Germany). 

### 2.3. SKALE 

The SKALE scoring system is based on the five parameters sex, KPS, ASA, location and peritumoral edema. It assigns a point value between 0 and 16, whereby surgery is initially recommended for patients with a score ≥8 (Table 1).

Peritumoral edema was analyzed in T2-weighted MRI and was considered to be severe in case of ratio of maximum edema ray on the maximum tumor diameter >1 or rated moderate with a ratio ≤1 (2 points) [16]. The SKALE score was calculated for the elderly cohort only.

### 2.4. Statistical Analysis

The cohort was dichotomized between younger and elderly patients with a cutoff at 70 years. Patient characteristics including gender, tumor grade according to the WHO classification, MIB-1 proliferation index and tumor volume were compared. To assess whether elderly patients profit from surgical treatment, we analyzed the perioperative course of KPS, major perioperative complications, length of clinical stay, intermediate care unit (IMC) stay, intensive care unit (ICU) stay, progression free survival (PFS) and the mortality rate. We further investigated the influence of individual preoperative SKALE parameters as well as total SKALE score on patient mortality.

Statistical analyses and figure design were performed using the JMP 14.0 software (SAS, Cary, NC, USA), GraphPad Prism 6 (GraphPad Software Inc., La Jolla, CA, USA) and Gimp2. Survival analyses were performed using Kaplan-Meier method with log-rank test. Nominal dichotomized variables were tested using Fisher’s exact test, while *t*-test was applied for ordinal distributed variables. After testing for normal distribution by using the Shapiro-Wilk-W-test we used the Mann-Whitney U test for non-normal distributed data. The Likelihood-ratio was used for our univariate analysis. A significance level of alpha < 0.05 was chosen for all tests.

## 3. Results

### 3.1. Patient Cohort

The cohort of 421 patients (male/female 134/287) underwent 447 surgeries for primary or recurrent meningioma resection in our neurosurgical department. 71 (=16.9%) of these patients were older than 70 years at the date of primary resection (male/female 37/34). Median age in our total cohort was 57 years (mean 56.6; range 16–86). In the elderly cohort, the median age was 73 years (mean 73.8; range 70–86).

**Figure 1:**
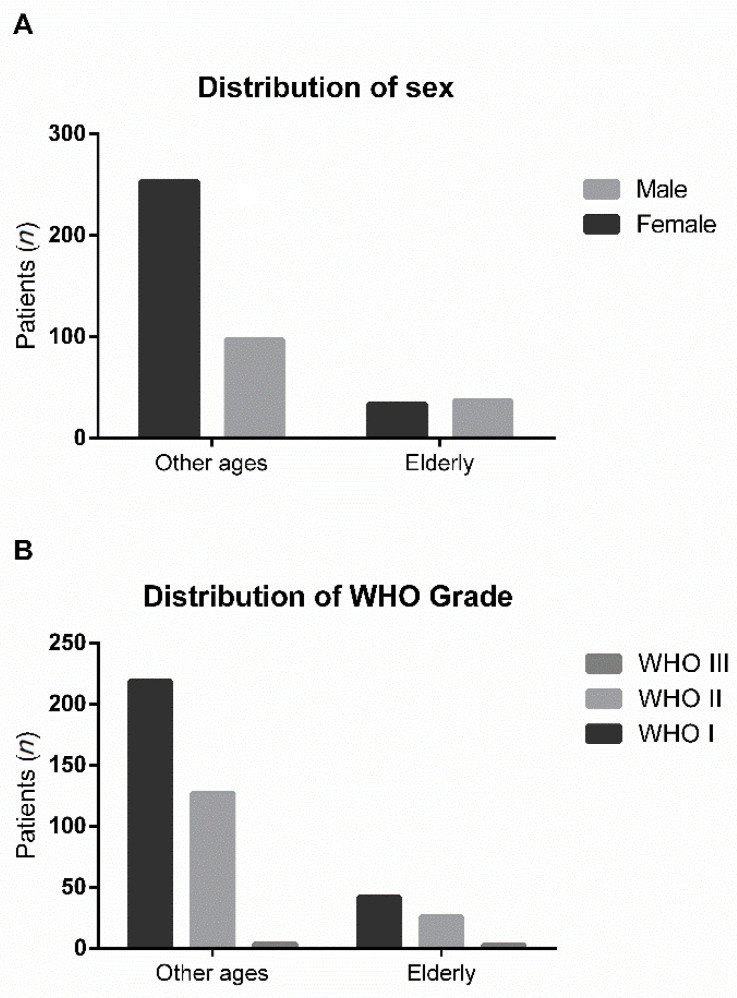
(**A**) Distribution of sex in younger and elderly patients. (**B**) WHO grade distribution in patients of other ages and elderly patients.

Female patients were predominantly represented in our younger cohort (72.3%). In contrast, in the elderly cohort only 47.9% of our patients were females (Figure 1A). This difference was statistically significant (Fisher’s exact test: *p* < 0.0001). 

Data on WHO grades were available for all patients. The distribution of WHO grades among primary meningiomas in the younger cohort (*n* = 350) was 219 WHO grade I (62.6%), 127 WHO grade II (36.3%) and 4 WHO grade III (1.1%). In our elderly cohort (*n* = 71), we identified patients with 42 WHO grade I (59.2%), 26 WHO grade II (36.6%) and three WHO grade III tumors (4.2%) (Figure 1B). This difference in WHO grade distribution was not statistically significant (Fisher’s exact test: *p* = 0.3400). Regarding MIB-1 proliferation index, we were also unable to detect any significant differences (younger: median 3%, range 1–25%, interquartile range (IQR) 3–5% vs. elderly: median 4%, range 1–40%, IQR 3–5.75%; *t*-test: *p* = 0.0638). No relevant differences were found with respect to tumor volumes (younger: median 22.5 cm^3^, range 0.3–214.6 cm^3^, IQR 7.8–49.4 cm^3^ vs. elderly: median 30.7 cm^3^, range 3.8–157.7 cm^3^, IQR 16.2–51.1 cm^3^; *t*-test: *p* = 0.2534).

### 3.2. Perioperative Morbidity

**Figure 2:**
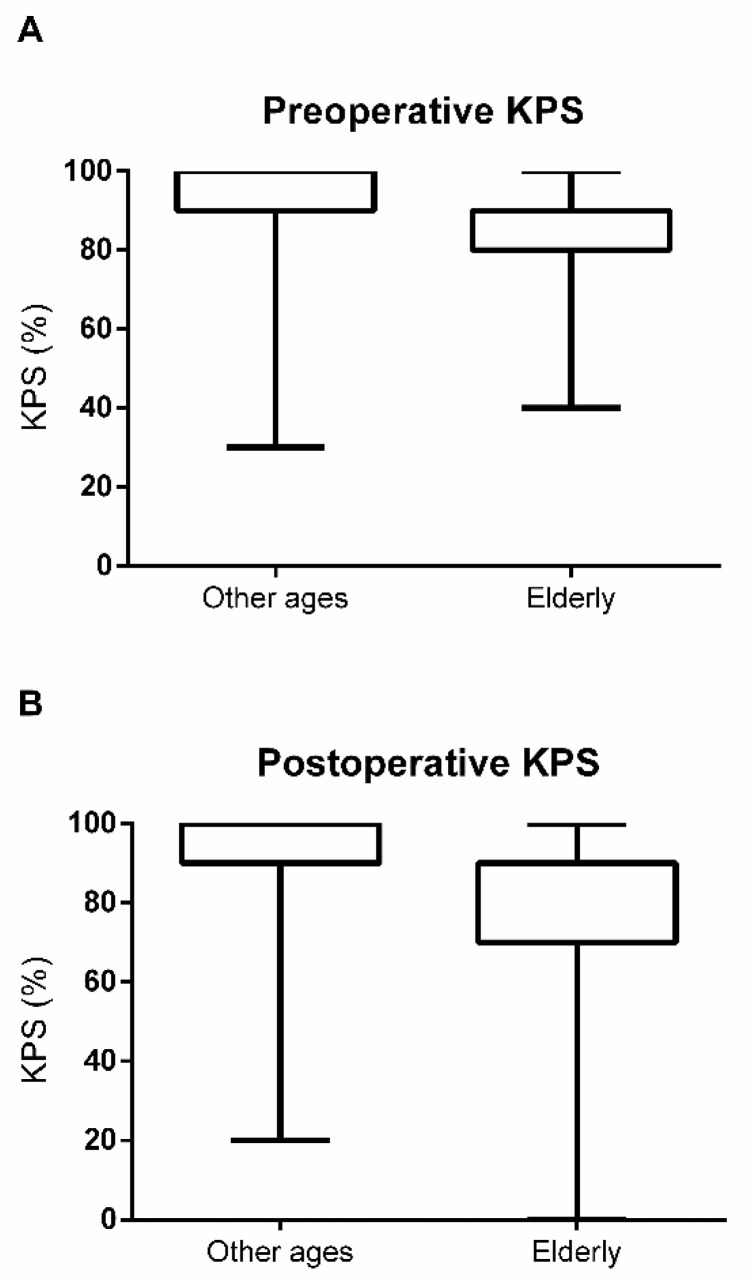
Comparison of younger and elderly patients regarding preoperative (**A**) and postoperative (**B**) Karnofsky Performance Scale (KPS).

**Figure 3:**
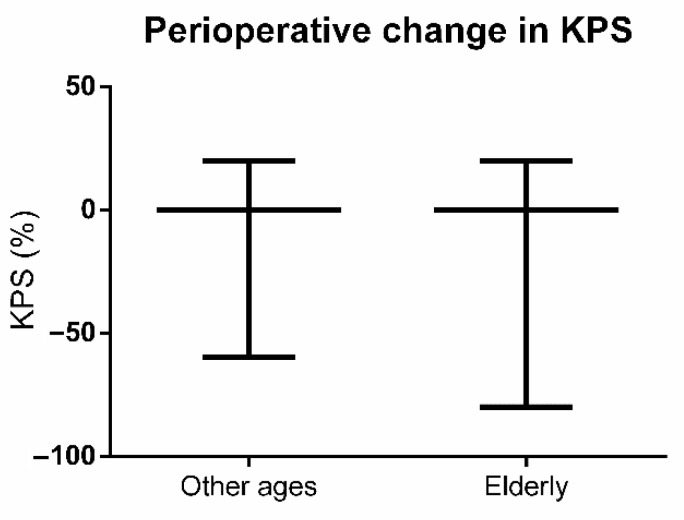
Perioperative Karnofsky Performance Scale (KPS) change in patients of other ages and elderly patients.

Perioperative KPS data were available for all patients. With regard to preoperative KPS, elderly patients had significantly lower KPS values (younger: median 90, range 30–100, IQR 90–100 vs. elderly: median 90, range 40–100, IQR 80–90; Mann-Whitney U test: *p* = 0.0003; Figure 2A). This finding persisted for the postoperative KPS (younger: median 90, range 20–100, IQR 90–100 vs. elderly: median 90, range 0–100, IQR 70–90; Mann-Whitney U test: *p* < 0.0001; Figure 2B). For perioperative KPS change, 13 out of 71 elderly patients (18.3%) experienced a deterioration compared to 45 of 350 younger patients (12.9%). This difference did not reach statistical significance (Mann-Whitney U test: *p* = 0.5848; Figure 3).

Postoperative length of stay in the elderly cohort was significantly extended (younger: median 8 days, range 2–124 days, IQR 7–13 days vs. elderly: median 11 days, range 4–44 days, IQR 8–14 days; *t*-test: *p* = 0.0297). Interestingly, no differences were found with respect to ICU stay (younger: median 1 day, range 0–121 days, IQR 1–2 days vs. elderly: median 1 day, range 1–35 days, IQR 1–6.5 days; *t*-test: *p* = 0.0916) and IMC stay (younger: median 2 days, range 0–13 days, IQR 1–4 days vs. elderly: median 3 days, range 1–25 days, IQR 2–7.75 days; *t*-test: *p* = 0.0991). 

With regard to postoperative complications, the elderly cohort did not receive more frequent re-craniotomy due to swelling or postoperative hemorrhage (14.7% vs. 15.4%; Fisher’s exact test: *p* = 0.5039). Similarly, a comparable number of revisions were performed due to wound infections (9.6% vs. 8.9%; Fisher’s exact test: *p* = 0.6414). Concerning pulmonary complications, we observed significantly more of those in our elderly cohort (pneumonia: 3.2% vs. 10%; Fisher’s exact test: *p* = 0.0202; pulmonary embolism: 6% vs. 12.7%; Fisher’s exact test: *p* = 0.0209).

However, we could not observe a higher incidence of sepsis in our elderly cohort (0.58% vs. 0%; Fisher’s exact test: *p* = 1.0000). Acute renal failure did not occur more frequently during the clinical stay in either patient cohort (0.3% vs. 1.4%; Fisher’s exact test: *p* = 0.3146).

### 3.3. Survival 

**Figure 4:**
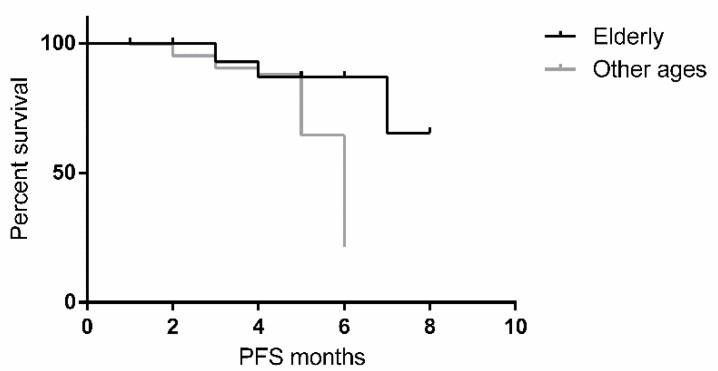
Kaplan-Meier curve showing the comparison of progression free survival (PFS) in patients of other ages and elderly.

Progression-free survival did not differ significantly between younger and elderly patients (mean 65.5 vs. 37.7 months; Log-rank test: *p* = 0.7500; Figure 4). Overall survival (OS) however, showed significantly worse prognosis for elderly patients but not tumor related (Supplementary Figure S1).

There was a statistical trend for higher perioperative mortality, defined as one-month mortality, in the elderly population (0.3% vs. 2.8%; Fisher’s exact test: *p =* 0.0751). Additional analyses of the Kaplan-Meier curves resulted in higher three-month (0.3% vs. 5.6%; Fisher’s exact test: *p* = 0.0033), 6-month (0.3% vs. 7%; Fisher’s exact test: *p* = 0.0006) and one-year mortality (0.3% vs. 8.5%; Fisher’s exact test: *p* < 0.0001) for elderly patients.

### 3.4. Influence of SKALE Score and Parameters

Complete SKALE data were available in all elderly patients. Balanced distribution of SKALE score was obtained for our elderly cohort (Table 2). Univariate analyses did not show an association between single SKALE parameters and postoperative outcome with the exception of tumor location. When comparing between critically and non-critically located tumors, there was a striking difference in one-year mortality rates (13% vs. 0%; Likelihood-ratio: *p* = 0.0190). Table 3 provides an overview of the SKALE parameters influence on the one-year mortality. 

**Figure 5:**
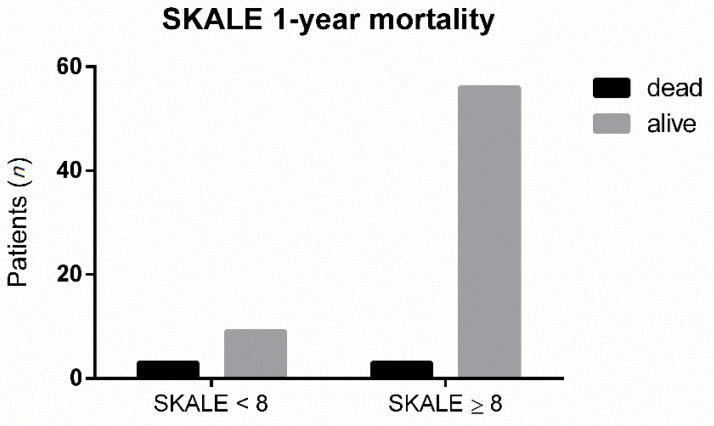
Comparison of SKALE <8 or SKALE ≥8 in terms of one-year mortality.

When dichotomizing elderly patients between total SKALE scores ≥8 and <8, a higher score was associated with significantly lower mortality at one year (5.1% vs. 25%%; Likelihood-ratio: *p* = 0.0479; Figure 5).

## 4. Discussion

### 4.1. Surgical Outcome

The aim of our study was to assess the outcome of elderly meningioma patients ≥70 years of age compared to a younger subgroup and to further evaluate the SKALE score as a decision-making aid in clinical practice. 

Initially, a potential bias in terms of patient selection could be excluded, since our elderly cohort did not contain a higher proportion of aggressive tumors or larger tumor volumes, which might be more complicated in surgical management. This finding is also supported by the fact that elderly patients had similar PFS compared to younger patients. 

Regarding morbidity, we were able to demonstrate that elderly patients face a considerable risk of postoperative deterioration. About one fifth of our patients had lower KPS scores postoperatively than preoperatively. In terms of KPS, Amano et al. reported similar perioperative courses between young and elderly patients ≥75 years [24]. This is consistent with the results of our study. However, a significantly lower postoperative KPS is observed in our elderly cohort, which is relevant concerning morbidity and quality of life and confirms the need for a careful patient selection.

Moreover, we were able to show a noticeable, prolonged length of hospitalization in our elderly cohort, and with this a higher rate of postoperative complications such as pneumonia and pulmonary embolism. This is in line with the findings of Zuo et al. who reported on postoperative pneumonia as a major complication in meningioma patients, especially at higher ages [25].

Although perioperative morbidity was high in our elderly patients, only a few experienced perioperative mortality. In 1995, Mastronardi et al. described a one-month mortality of 29% for patients >80 years [26]. This contrasts with a one-month mortality rate of 0% reported by Sacko et al. in 2007 and 3.9% for the cohort of Konglund et al. in 2013 [21]. We assume that stated differences in surgical mortality might be explained by advanced surgical and perioperative techniques, more stringent patient selection and different age distribution. 

However, the one-year mortality rate in our cohort was higher for the elderly subgroup. This is in line with the results of Brokinkel et al., who compared the outcome of patients ≥65 years with a younger cohort in a large-scale study in 2017 [14]. Contrary to this, Poon et al. could not confirm statistical significance but also reported on a trend towards higher one-year mortality for patients ≥65 years (4.3% vs. 1.1%) [11].

Referring to the considerably higher three-month and six-month mortality in our elderly cohort, a substantially increased operative risk for patients ≥70 years of age might be assumable. This needs to be kept in mind when advising patients toward surgical intervention. Furthermore, it should be considered that patients with higher age and asymptomatic meningioma might not benefit from surgery. The lower preoperative KPS in our elderly cohort might be interpreted as preselection towards a higher proportion of symptomatic patients.

Especially in patients with higher-grade meningiomas, the promising data regarding drug therapy with VEGF inhibitors, mTOR inhibitors or tyrosine kinase inhibitors should be considered. This therapy could gain importance in the future and represent a reasonable alternative for patients without a surgical therapy approach [27,28].

### 4.2. SKALE Score

Since the postoperative course of elderly meningioma patients was very heterogenous in our cohort, a valid score to estimate postoperative outcome seems to be a valuable tool for better patient selection. We confirmed that the SKALE score [16] might be a suitable tool to guide surgical decision making, though previously published studies produced conflicting data in terms of validity [15,16,21,29].

First described by Sacko et al. in 2007, SKALE was introduced as a grading system for meningioma patients ≥80 years. It is based on parameters found to be significant in terms of one-year mortality [16]. Schul et al. could not validate the significance of the SKALE score regarding survival in a series of 164 patients ≥65 years. Only concomitant disease and ASA score were found to be significant in single factor analysis [15]. Yet, Konglund et al. in 2013 concluded that SKALE, compared to three other scoring systems, correlated best with the one-year mortality of patients between 80 and 90 years. Patients with a SKALE score of >8 had a 1-year mortality of 14.3%, compared to 50% for =8 and 56.3% for <8. Statistical analysis verified prolonged survival for SKALE scores >8. In a univariate analysis of the individual parameters, sex, ASA score and peritumoral edema were found to be significant [21]. Interestingly, Delgado-Fernández et al. concluded in 2018 that SKALE has the highest predictive value in terms of morbidity and mortality compared with previously investigated scores. Again, the cutoff was set at a score of ≥8 according to the original description by Sacko et al. Regarding the individual parameters, it was found that a low ASA score and a high preoperative KPS were decisive with regard to the postoperative outcome [29].

For the single SKALE parameters, we could only verify the location as a statistically relevant prognostic parameter. This seems comprehensible, since tumor location at the skull base, in eloquent areas or close to large vessels are surgically more challenging, with an elevated risk of complications.

With respect to one-year mortality, our data aligns with the results of Konglund et al. and Sacko et al. Concerning the cut-off score, we demonstrated that patients with a score of <8 are at high risk of mortality, corresponding to 25% in our cohort [16,21]. Therefore, we would support Sacko et al. and Delgado-Fernández et al., setting the SKALE score cut-off at ≥8 [16,29]. 

### 4.3. Limitations of Our Study

The major limitation of this study is the potential selection bias of patients before operation, since only surgically treated patients have been included. Conservatively or radio-surgically treated patients were not included. Another important limitation is the retrospective design of our study.

## 5. Conclusions

Our data suggest that elderly meningioma patients are exposed to a substantially higher risk of morbidity and mortality compared to younger patients when undergoing surgical treatment. Nevertheless, meningioma surgery in elderly patients seems reasonable since individual outcomes show great variety. The SKALE score is not reliable in its single components, yet the overall score gives a valuable estimate of the postoperative prognosis. We recommend further prospective studies on the validity of SKALE and other scoring systems to confirm relevance in clinical practice.

## Figures and Tables

**Table 1:** Summary of the SKALE single parameters and corresponding score value [16].

		Score	
	0	2	4
Sex	M	F	-
KPS	≤50	60–70	≥80
ASA	IV	III	I or II
Location	Critical	Not critical	-
Edema	Severe	Moderate	No edema

KPS = Karnofsky Performance Status Scale; ASA = American Society of Anesthesiology class.

**Table 2:** Distribution of the SKALE score in our elderly cohort.

Score	Amount	Share
2	1	1.4%
4	5	7%
6	6	8.5%
8	12	16.9%
10	22	31%
12	17	23.9%
14	6	8.5%
16	2	2.8%

**Table 3:** Single factor analysis of the SKALE parameters regarding their influence on one-year mortality.

	*n*	1-Year	*p*
male	37	13.5%	0.0943
female	34	2.9%	
KPS ≤ 50	5	40%	0.0711
KPS 60–70	8	0%	
KPS ≥ 80	58	6.9%	
ASA IV	1	0%	0.8930
ASA III	38	7.9%	
ASA I-II	32	9.4%	
Loc. critical	46	13%	0.0190
Loc. not critical	25	0%	
Severe edema	27	11.1%	0.7921
Moderate edema	26	7.7%	
No edema	18	5.6%	
SKALE ≥ 8	59	5.1%	0.0479
SKALE < 8	12	25%	

## Data Availability

The data presented in this study are available on request from the corresponding author. The data are not publicly available due to ethical restrictions.

## Supplementary Materials

The following are available online at https://www.mdpi.com/article/10.3390/jcm10091820/s1, Figure S1, Kaplan-Meier curve showing the comparison of overall survival (OS) in patients of other ages and elderly
