# Supplementary material for: Meningioma Surgery in Patients ≥70 Years of Age: Clinical Outcome and Validation of the SKALE Score
Source: J Clin Med. 2021 Apr 22;10(9):1820. doi: 10.3390/jcm10091820 (PMC8122404; doi:10.3390/jcm10091820)
Supplement: Supplementary file 1 [file jcm-10-01820-s001.zip › jcm-1172290-supplementary.pdf]

Supplementary Figure

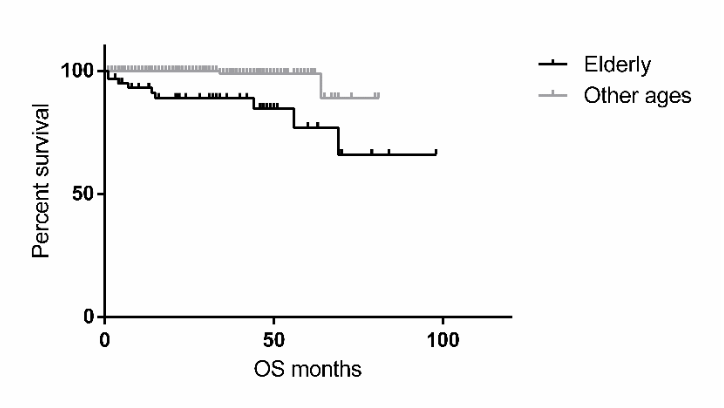

Figure S1. Kaplan-Meier curve showing the comparison of overall survival (OS) in patients of other ages and elderly (mean 58.4 vs. 63.5 months; Log-rank test: $p < 0.0001$). This finding is not surprising, given the fact that life expectancy in general is shorter for a person ≥70 years.
